# Factors associated with anaemia and iron deficiency among women of reproductive age in Northeast Thailand: a cross-sectional study

**DOI:** 10.1186/s12889-020-8248-1

**Published:** 2020-01-28

**Authors:** Jutatip Jamnok, Kanokwan Sanchaisuriya, Pattara Sanchaisuriya, Goonnapa Fucharoen, Supan Fucharoen, Faruk Ahmed

**Affiliations:** 1 Medical Science Program, Graduate School, Khon Kaen University, Khon Kaen, Thailand; 2 Centre for Research and Development of Medical Diagnostic Laboratories (CMDL), Faculty of Associated Medical Sciences, Khon Kaen University, Khon Kaen, Thailand; 3 Faculty of Public Health, Khon Kaen University, Khon Kaen, Thailand; 4 Public Health, School of Medicine, Griffith University, Gold Coast, Australia; 5 Menzies Health Institute Queensland, Griffith University, Gold Coast, Australia

**Keywords:** Anaemia, Iron deficiency, Thalassemia

## Abstract

**Background:**

Anaemia and iron deficiency (ID) affect women of reproductive age globally and considered to be a major public health problem in developing countries. This study determines the prevalence of anaemia and ID among women of reproductive age in urban northeast Thailand and examined the relative contribution of various risk factors to anaemia and ID in this population.

**Methods:**

Three hundred ninety-nine non-pregnant women, aged 18–45 years, from three universities in northeast Thailand participated in this cross-sectional study. Selected socio-demographic, history of blood loss, usual consumption of red meat and tea/coffee, and anthropometric data were collected. Complete blood count including haemoglobin (Hb) concentration, serum ferritin (SF), C-reactive protein (CRP), and thalassemia were determined. Multiple logistic regressions were applied to identify the risk factors of anaemia and ID.

**Results:**

Overall, 370 participants were included for data analyses after excluding women with severe/intermedia thalassemia diseases and/or those with positive serum CRP. The prevalence of anaemia, ID, and iron deficiency anaemia (IDA) were 28.4, 28.4, and 13.2%, respectively. Women with thalassemia had a higher prevalence of anaemia but a lower prevalence of ID than the women without thalassemia. By multiple regression analysis, ID [adjusted OR (AOR) = 4.9, 95% CI = 2.8–8.3], two α-gene defects (AOR = 8.0, 95% CI = 3.0–21.3) and homozygous Hb E (AOR = 8.5, 95% CI = 3.0–24.3) were identified as the potential risk factors of anaemia. Further, the odds of ID were significantly higher among women who donated blood within the past 3 months (AOR = 6.7, 95% CI = 2.8–16.3), and had moderate to a high amount of blood loss during menstruation (AOR = 2.2, 95% CI = 1.3–3.9).

**Conclusion:**

This study found a relatively high but differential prevalence of anaemia and ID among women of reproductive age with or without thalassemia. Only homozygous Hb E and two α-gene defects of thalassemia types and ID were the main factors contributing to anaemia. Recent blood donation, and moderate to a high amount of blood loss during menstruation were potential risk factors of ID in this population.

## Background

Anaemia affects more than 500 million women of reproductive age globally and it is considered to be a major public health problem in developing countries [1]. Anaemia is associated with various physiologic consequences including impaired tissue oxygen delivery, weakness, fatigue; and loss of productivity from reduced work capacity, cognitive impairment and increased susceptibility to infections [2] and which also imposes a substantial economic burden on an individual [3]. Anaemia has also been reported to contribute to maternal morbidity and mortality [4, 5].

Causes of anaemia are multiple and complex, but iron deficiency (ID) is considered to be the major cause of anaemia, especially among women of reproductive age due to limited intake of iron-rich foods along with poor bioavailability, and increased requirement associated with menstruation [6–9]. While iron supplementation is widely practised to control anaemia, especially among women of reproductive age, the current evidence suggests that there has been a limited impact of iron supplementation, under the programmatic condition, on reducing the anaemia in most of the developing countries [10]. Therefore, identifying the underlying causes of anaemia is crucial for developing an effective anaemia control program. Among other factors that contribute to anaemia include malaria, hookworm infestation, chronic infection, thalassemia and hemoglobinopathies, and other nutritional deficiencies such as folic acid, vitamin B_12_ and vitamin A [6, 11, 12].

The World Health Organization (WHO) global health observatory data repository on anaemia in 2016 has reported 31.8% prevalence of anaemia among women of reproductive age in Thailand and is considered to be a moderate public health problem [13]. In Thailand and other Southeast Asian countries, thalassemia and hemoglobinopathies are highly prevalent and a significant contributor to anaemia [6, 14, 15]. Northeast Thailand is an area where thalassemia is highly prevalent, and previous studies have shown that thalassemia and haemoglobinopathies are the major causes of anaemia among adolescents [16] and pregnant women [14] in rural Northeast Thailand. It is important to note that the clinical severity of different genotypes of thalassemia varies widely ranging from asymptomatic to severe form of anaemia [17]. Further, subjects with some genotypes of thalassemia carrier may also exhibit iron overload [18]. Therefore, it is imperative to assess the extent to which different thalassemia genotypes contribute to the prevalence of anaemia in this population. As iron is a pro-oxidant [19] and an excess amount can be harmful, it is important to identify the risk factors of anaemia before considering blanket iron supplementation program. Therefore, the present study was designed to determine the prevalence of anaemia, ID and thalassemia genotypes among women of reproductive age in urban northeast Thailand and identify the relative contribution of various risk factors associated with anaemia and ID in this population.

## Methods

### Study design and subjects

This cross-sectional study was conducted among reproductive-age women (*n* = 399), aged between 18 and 45 years, who were recruited from two universities in Khon Kaen and one university from Udon Thani provinces, northeast Thailand. The subjects who were pregnant, had any sign of chronic diseases, had a surgery or blood loss from an accident, and who took iron tablets within 3 months preceding the data collection were excluded from the study. The study protocol was approved by the ethics committee (EC) of Khon Kaen University, Thailand. The study was carried out between January and May 2017.

The sample size for the study was calculated based on the prevalence of anaemia observed in a previous study among women of reproductive age in Northeast Thailand [20]. Considering 37% prevalence of anaemia with an estimated 95% confidence interval of 31.4–42.5 [20], 358 subjects were required to achieve a valid estimate of the prevalence of anaemia. After including a 10% allowance to compensate for sample loss during data collection, the study required 394 women.

### Questionnaire and sample collection

Convenience sampling was applied to recruit study participants. The project was announced through the communication media, such as poster around the university campus and using Facebook. Besides, one of the researchers visited different classes to announce the project**.** The interested prospective participants were requested to contact the principal researcher directly. The purpose of the study was explained to all eligible participants and those who agreed to participate in the study were requested to sign on a consent form. Those who met the selection criteria were asked to complete a self-administered questionnaire. The questionnaire consists of socio-demographic information (age, education level, occupation, family income) and history of blood loss (history of blood donation, accident and/or surgery, the length of menstrual bleeding, amount of blood loss per day). Amount of blood loss per day was assessed by self-estimation. In comparison with an 80-ml glass of water, blood loss < 1 glass was considered low. Conversely, blood loss > 1 glass was considered moderate to high. The participants were also asked to report their body weight and height, and usual dietary behaviour regarding the consumption of red meat and tea/coffee drinking. The participants reported “yes” if they usually eat red meet or drink tea/coffee at least once a week, or “no” if they don’t. Five millilitres of blood samples were collected from each participant following the completion of the questionnaire.

### Laboratory analysis

Complete blood count (CBC) including haemoglobin (Hb) concentration was determined using the Sysmex Hematology Analyzer (XN-1000, Sysmex, Kobe, Japan). Serum ferritin (SF) concentration, as a measure of iron status, was determined by the chemiluminescent micro-particle immunoassay (CMIA) (Abbott; ARCHITECT i2000SR Immunoassay, USA). The serum C-reactive protein (CRP), a marker of the presence of infection and/or inflammation [21, 22], was measured using CRP Latex Test Kit (Plasmatec Co., Dorset, UK). The results of CRP were interpreted by agglutination.

An automated capillary zone electrophoresis (Capillarys, Sebia, France) technique was used to determine the thalassemia (Hb type and the corresponding fraction). Beta-thalassemia was diagnosed in participants with mean corpuscular volume (MCV) <  80 fL and Hb A_2_ > 4% [23]. Deletional, non-deletional, and mutations of α-thalassemia, most common in Southeast Asia countries, including α^0^-thalassemia (SEA and THAI deletions), α^+^-thalassemia (3.7 kb and 4.2 kb deletions), Hb Constant Spring (Hb CS), and Hb Pakse (Hb Ps) were identified using PCR-based techniques [24–26].

### Statistical analysis

In the present study sample, we have identified 21 women with inflammation or infection judged by positive serum CRP. As the presence of inflammation or infection is known to influence serum ferritin levels [22], these women were excluded from the analysis. Besides, 8 women with severe/intermedia thalassemia diseases (i.e. 3 with β-thalassemia/Hb E disease, 2 with Hb H disease, 3 with CSEABart’s or EABart’s disease) who exhibited extremely low Hb and high SF levels, were also excluded from the analyses. Thus, a total of 370 women were included in the statistical analyses.

Anaemia was defined as a Hb concentration < 12.0 g/dL [27]. Iron deficiency was defined as SF concentration < 15.0 μg/L [27]. IDA was defined as a Hb concentration < 12.0 g/dL and a SF concentration < 15.0 μg/L [27]. Prevalence of anaemia, ID and IDA were summarized as percentage with 95% confidence interval (CI).

The normality of the distribution of data was checked by the Kolmogorov-Smirnov goodness-of-fit test. The data of Hb concentrations and red blood cell count (RBC) were negatively skewed, while SF concentrations were positively skewed. Therefore, the medians with interquartile ranges were used to describe these data. For multiple comparison of continuous variables, the Kruskall-Wallis one-way ANOVA was applied. Subsequent comparison between 2 independent groups was tested with the Mann-Whitney U test.

Multiple logistic regression was applied to identify the risk factors associated with anaemia and ID. For anaemia, the independent variables included iron status, different types of thalassemia, body mass index (BMI), age, education level, occupation, family income, history of recent blood donation, the length of menstruation, amount of blood loss per day during menstruation, red meat consumption and tea/coffee drinking. Types of thalassemia were categorized into 7 groups; i.e. non-thalassemia, one α-gene defect, two α-gene defects, Hb E trait, Hb E trait with one α-gene defect, Hb E trait with two α-gene defects and homozygous Hb E. For ID, the independent variables were BMI, age, education level, occupation, family income, history of blood donation, and the length of menstruation, amount of blood loss per day during menstruation, red meat consumption and tea/coffee drinking. Each explanatory variable was initially tested with a univariate model to select candidate variable (*p* < 0.25) to include in the multiple logistic regression analysis. The SPSS (version 25; SPSS Inc., Chicago) was used for statistical testing; and *p*-value < 0.05 was considered the statistical significance.

## Results

Of the 370 participants, about 30% were adolescents (aged 18–19 years). Nearly 90% of the participants were students with a large majority (> 80%) studying a Bachelor’s degree. Only 14.6% of the women had a low family income (< 10,000 Baht). Nine per cent of the participants were either overweight or obese. Over two-thirds of the participants had menstruation for 3–5 days. Forty-three per cent of the participants reported a moderate to a high amount of blood loss per day during menstruation. On the other hand, 14.6% of the participants had no idea about the estimated amount of blood loss during menstruation. Eight per cent of the participants donated blood in the 3 months preceding the interview. The usual dietary pattern indicated that about 82% of the participants consume red meat and 75% drink tea and/or coffee (Table 1).

**Table 1:** Socio-demographic characteristics, nutritional status, history of blood loss, and dietary practice of the study participants

Socio-demographic characteristics	n	%
Age (year)		
18–19	111	30.0
20–24	209	56.5
≥ 25	50	13.5
BMI (kg/m^2^)		
Underweight: < 18.5	78	21.1
Normal: 18.5–24.9	259	70.0
Overweight: 25.0–29.9	28	7.6
Obese: ≥ 30	5	1.4
Education Level		
High school	11	3.0
Bachelor’s degree studying	312	84.3
Master’s degree or Doctorate degree	47	12.7
Occupation		
Student	331	89.5
Teacher/Researcher/Medical Personal	39	10.5
Family income (Baht/month)		
< 10,000	54	14.6
10,000 – 20,000	149	40.3
20,000 – 30,000	58	15.7
> 30,000	101	27.3
Blood loss history		
Blood donation in recent 3 months		
No	335	90.5
Yes	31	8.4
Length of menstruation		
< 3 days	29	7.8
3–5 days	260	70.3
> 5 days	77	20.8
Blood loss during menstruation/day^a^		
Low	143	38.6
Moderate to high	160	43.2
No idea	54	14.6
Dietary practice		
Red meat (beef) consumption		
No	64	17.3
Yes	302	81.6
Drink tea/coffee		
No	93	25.2
Yes	276	74.8

^a^Self-estimation by comparing with a glass of water (Low: < 80 ml, Moderate to High: ≥ 80 ml)

Of the 370 participants, 28.4% had anaemia, 28.4% had ID, and 13.2% had IDA. Among 227 participants with thalassemia carrier, 32.2% had anaemia, 24.2% had ID, and 11.5% had IDA. Among 143 participants without thalassemia, 22.4% had anaemia, 35.0% had ID, and 16.1% had IDA (Table 2).

**Table 2:** Prevalence of anaemia, ID, and IDA among women of reproductive age in northeast Thailand

	n	Prevalence (%)	95% CI
Anaemia			
All subjects (*n* = 370)	105/370	28.4	23.8–33.0
Thalassemia (*n* = 227)	73/227	32.2	26.0–38.3
Non-thalassemia (*n* = 143)	32/143	22.4	15.4–29.4
ID			
All subjects (*n* = 370)	105/370	28.4	23.5–32.7
Thalassemia (*n* = 227)	55/227	24.2	18.9–29.5
Non-thalassemia (*n* = 143)	50/143	35.0	27.3–42.7
IDA			
All subjects (*n* = 370)	49/370	13.2	9.7–16.5
Thalassemia (*n* = 227)	26/227	11.5	7.1–15.9
Non-thalassemia (*n* = 143)	23/143	16.1	9.8–22.4

*CI* Confidence interval

Table 3 shows the distribution of thalassemia types and the corresponding median values of red blood cell (RBC), Hb and SF. Overall, 61.4% of women were found to carry thalassemia genes. The most common types were Hb E trait (25.7%) and one α-gene defect (14.3%). Comparing with the non-thalassemia group, there were significantly higher RBC counts in the participants with any genotypes of thalassemia. On the other hand, Hb concentrations were significantly lower among participants with two α-gene defects, Hb E trait, Hb E trait with two α-gene defects, and homozygous Hb E. Comparing with the non-thalassemia group, the participants with homozygous Hb E, two α-gene defects, and one α-gene defect had significantly high SF concentrations.

**Table 3:** Proportions of thalassemia types and the corresponding median values of RBC, Hb and SF concentrations among women of reproductive age in northeast Thailand

Thalassemia type	n	%	RBC (× 10^12^/L)	IQR	Hb (g/dL)	IQR	SF (μg/L)	IQR
Non-thalassemia	143	38.6	4.69	4.43–4.95	12.8	12.1–13.5	24.8	8.6–43.4
One α-gene defect^a^	53	14.3	4.98*	4.79–5.23	12.5	12.1–13.1	34.1*	15.6–58.3
Two α-gene defects^b^	22	5.9	5.54*	5.10–5.80	11.8*	10.4–12.3	48.4*	29.2–63.2
Hb E trait	95	25.7	5.21*	4.88–5.42	12.4*	11.8–13.0	27.9	13.9–50.4
Hb E trait with one α-gene defect	28	7.6	4.96*	4.79–5.15	12.5	12.0–13.1	23.7	11.3–52.2
Hb E trait with two α-gene defects	10	2.7	5.59*	5.23–5.93	12.1*	11.6–12.4	24.8	11.9–97.6
Homozygous Hb E	19	5.1	6.10*	5.51–6.34	11.6*	11.0–12.4	44.4*	33.0–64.2

*RBC* Red blood cell count, *IQR* Interquartile range, *Hb* Haemoglobin, *SF* Serum ferritin

^a^Including α^+^-thalassemia trait, Hb CS trait

^b^Including homozygous α^+^-thalassemia, homozygous Hb CS, α^0^-thalassemia trait, and β-thalassemia trait

^*^p-value < 0.05 for the difference between non-thalassemia and thalassemia type (Mann-Whitney U test)

Factors associated with anaemia were examined using multivariate logistic regression analysis (Table 4). Only two α-gene defects and homozygous Hb E thalassemia, and iron status were statistically significant. The participants with ID were 4.9 times more likely to suffer from anaemia compared with the participants without ID (AOR: 4.9, 95% CI: 2.8–8.3). Among the thalassemia, only those with homozygous Hb E (AOR: 8.5, 95%CI: 3.0–24.3) and two α-gene defects (AOR: 8.0, 95% CI: 3.0–21.3) had a significant association with anaemia.

**Table 4:** Logistic regression analysis for anaemia and ID among women of reproductive age in northeast Thailand

	Variable	n	Adjusted OR* (95%CI)		*p*-value
Anaemia	Iron status				
	Non-ID (reference)	265			
	ID	105	4.9	(2.8–8.3)	< 0.001
	Thalassemia type				
	Non-thalassemia (reference)	143			
	One α-gene defect^a^	53	1.1	(0.5–2.6)	0.754
	Two α-gene defects^b^	22	8.0	(3.0–21.3)	< 0.001
	Hb E trait	95	1.9	(1.0–3.5)	0.055
	Hb E trait with one α-gene defect	28	0.9	(0.3–2.5)	0.793
	Hb E trait with two α-gene defects	10	2.8	(0.7–11.7)	0.149
	Homozygous Hb E	19	8.5	(3.0–24.3)	< 0.001
ID	Drink coffee/tea				
	No (reference)	93			
	Yes	276	1.7	(0.9–3.3)	0.113
	Blood donation in recent 3 months				
	No (reference)	335			
	Yes	31	6.7	(2.8–16.3)	< 0.001
	Blood loss during menstruation/day				
	Low (reference)	143			
	Moderate to high	160	2.2	(1.3–3.9)	0.003

*ID* Iron deficiency

^a^Including α^+^-thalassemia trait, Hb CS trait

^b^Including homozygous α^+^-thalassemia, homozygous Hb CS, α^0^-thalassemia trait, and β-thalassemia trait

*For anaemia, adjusted for length of menstruation and blood donation in recent 3 months; For ID, adjusted for BMI and length of menstruation

A similar analysis was carried out to identify the risk factors of ID (Table 4). The women who donated blood within the three months preceding the interview were 6.7 times more likely to suffer from ID (AOR: 6.7, 95% CI: 2.8–16.3). The participants with moderate to a high amount of blood loss/day during menstruation had significantly higher OR (AOR: 2.2, 95% CI: 1.3–3.9) when compared to the women who had low-level daily blood loss during menstruation. The participants who usually consume tea or coffee were 1.7 times more likely to suffer from ID compared with the participants who do not consume tea or coffee (AOR: 1.7, 95% CI: 0.9–3.3). However, the result was not statistically significant.

## Discussion

The present study reports the prevalence and the risk factors of anaemia and ID among women of reproductive age in northeast Thailand. Overall, 28% of the women were affected by anaemia. A study conducted in central Thailand reported 21% of anaemia among women of reproductive age studying in a university [28]. The difference in the prevalence of anaemia might be due in part to the difference in the prevalence of thalassemia; with more than 60% of the participants in the present study had thalassemia, while the prevalence of thalassemia in central Thailand study was reported to be 33–38% [29, 30]. Nonetheless, food consumption behaviour, which differs from region to region, may also account for the difference in anemia prevalence. Of note, the high prevalence and complexity of thalassemia observed in our study, which was very similar to that were reported in previous studies on adolescent, pregnant women, and vegetarians community in northeast Thailand [14, 16, 31], thus indicating the presence of heterogeneity of thalassemia and hemoglobinopathies in the region.

In the present study, overall, over a quarter of the study participants had ID and 13.2% were found to have IDA. A very similar prevalence of ID was reported in earlier studies in adolescent girls aged 15–17 years in rural northeast Thailand [16] and in women of reproductive age in Lao, PDR [32]. When compared with the women without thalassemia, there was a significantly higher prevalence of anaemia but a lower prevalence of ID and IDA among women with thalassemia. Further, the present study also showed a significantly higher RBC counts but lower Hb concentrations in women with different genotypes of thalassemia compared with that of the women without thalassemia. Thalassemia is a genetic Hb disorder that results in decreased or defective Hb production, thus leading to anaemia [17]. In thalassemia carriers, the bone marrow attempts to compensate for the low Hb level by increasing RBC production, hence, leading to an increased number of RBC in blood circulation [33, 34].

Our results also revealed significantly higher SF concentrations among women with homozygous Hb E, two α-gene defects, and one α-gene defect. As for this study, an earlier study showed that SF in α^0^-thalassemia trait and β-thalassemia trait were significantly higher than that of normal controls [18]. Besides, studies have shown increased gastrointestinal absorption of iron in non-transfusion-dependent thalassemia patients and thus develop increased body iron loads due to ineffective erythropoiesis and hypoxia dependent hepcidin downregulation [35–37]. It is noteworthy that in the present study, only five women had iron overload (SF concentrations > 150 μg/L), four of them had thalassemia (data not shown).

The preliminary results of multiple logistic regression analysis for anaemia indicated that thalassemia (OR: 2.0, 95% CI: 1.1–3.5) and ID (OR: 3.7, 95% CI: 2.0–6.7) were the main risk factors of anaemia in this population (data not shown). Since the severity of clinical manifestation of different genotypes of thalassemia relates to the amount of globin chain generated and stability of remaining chains present in excess [17, 33], in the final regression model, we included different types of thalassemia as independent variables, instead of total thalassemia, along with ID. After adjusting for the potential confounders, we found that the women with homozygous Hb E and carriers of two α-gene defects were 8.0 to 8.5 times more likely to develop anaemia. None of the other types of thalassemia was significantly associated with anaemia. The findings of this study are in agreement with the findings of the previous study conducted among pregnant women in northeast Thailand [14]. Similarly, Cambodian studies also found that homozygous Hb E was the most significant predictor of Hb level among children and women of reproductive age [15, 38]. Thus, our findings confirm previous study results that different genotypes of thalassemia exhibit variation in the risk of developing anaemia. Since both homozygous Hb E and carriers of two α-gene defects are prevalent in the region [14, 16, 31], they need to be taken into consideration in the planning of anaemia control program in the region. We also found that women with ID were 5 times more likely to develop anaemia when compared to women with normal iron status. The findings of our study are similar to that was observed in a previous study on pregnant women in northeast Thailand [14].

Since ID was identified as an independent risk factor of anaemia in this study population, which can be preventable by an appropriate intervention such as nutrition education with or without iron supplementation, we carried out multiple logistic regression analysis to identify the risk factors of ID. After adjusting for potential confounders, recent blood donation and amount of blood loss during menstruation were identified as the independent risk factors of ID in this population. We found that the women who donated blood within 3 months preceding the interview were 6.7 times more likely to develop ID than the women who did not. Our finding corroborates with the findings of a previous study that female blood donors had low iron stores and high prevalence of ID compared with the females who did not [39]. The present study also revealed that the women who had moderate to a high level of blood loss during menstruation were 2.2 times more likely to have ID than the women who had low-level blood loss during menstruation. A study among women of childbearing age in the UK also reported that menstrual blood loss was a significant predictor of iron status [40].

Although evidence from experimental studies have shown that the polyphenolic-containing compounds including tea and coffee are the potent inhibitors of non-heme iron absorption [41, 42], whether tea/coffee drinking has a negative impact on the body iron stores is controversial. As for other cross-sectional studies [43–45], we found no association between tea/coffee drinking and ID. On the contrary, the association between tea drinking and poor iron status has been reported [46]. A case with IDA due to excessive green tea consumption has also been recently demonstrated [47]. The negative impact of tea/coffee drinking on iron status may depend on the lifestyles and consumption habits, such as types and amounts of tea/coffee, frequency and timing of drinking as well as the consumption of other dietary modifiers, such as plant-based diet [41].

It is important to note that among the non-thalassemia group (*n* = 143), 32 women were anaemic of which, 23 had IDA. The reasons for anaemia in the rest of the women (*n* = 9) could not be explained by ID. Besides deficiency of iron, deficiency in other micronutrients, such as vitamin B12, folate, vitamin A, and riboflavin also cause anaemia [48]. These women may have other micronutrient deficiencies, which needs further investigation. Another possible cause could be chronic inflammation as the other inflammation markers were not determined.

The present study has some limitations. Firstly, the majority of participants were well educated and lived in urban areas with a relatively higher socio-economic background. Because the study did not include women who are not attending/working in the universities, the study samples may not be representative of the wider population from which they were drawn. Therefore, the results cannot be taken as representative of all women of reproductive age in urban northeast Thailand. Secondly, as a cross-sectional study, it should be bear in mind that it may not be possible to infer that recent blood donation and amount of blood loss during menstruation preceded the onset of ID. Thirdly, we used the self-reported nature of the questionnaire for collecting information on blood loss during menstruation, which may have introduced some bias. Fourthly, the data on dietary practice regarding the consumption of red meat and tea/coffee drinking lack information on the frequency of consumption and the actual amount of consumption. However, the findings of the present study are in line with previous studies, indicating that anaemia and ID remain a health burden in reproductive-age women, even well-educated.

## Conclusion

The present study revealed that a significant proportion of urban well-educated women with a relatively high socio-economic background in northeast Thailand had anaemia. While thalassemia is widely prevalent, ID was also a significant problem in this population. Our results suggest that both thalassemia and ID are the major risk factors of anaemia. However, among the various genotypes of thalassemia, only homozygous Hb E and carriers of two α-gene defects were independently associated with anaemia. Further, recent blood donation and moderate to a high amount of blood loss during menstruation were identified as the potential risk factors of ID in this population. These findings have important implications for the development of effective health promotion program for the prevention and control of anaemia in northeast Thailand. The present study emphasizes the need for comprehensive intervention strategy including creating awareness among female blood donors and individuals with high menstrual losses to improve iron status and thus reducing the anaemia.

## Data Availability

We declare that we can provide all materials, questionnaires, and data are available from the corresponding author on reasonable request.

## Abbreviations

AOR: Adjusted OR
BMI: Body mass index
CBC: Complete blood count
CMIA: Chemiluminescent micro-particle immunoassay
CRP: C-reactive protein
EC: Ethics committee
Hb CS: Hb Constant Spring
Hb Ps: Hb Pakse
Hb: Haemoglobin
ID: Iron deficiency
IDA: Iron deficiency anaemia
IQR: Interquartile range
MCV: mean corpuscular volume
RBC: Red blood cell count
SF: Serum ferritin
WHO: World Health Organization

## References

[1] World Health Organization (2015). The global prevalence of anaemia in 2011.

[2] Haas JD, Brownlie T (2001). Iron deficiency and reduced work capacity: A critical review of the research to determine a causal relationship. J Nutr.

[3] Horton S, Ross J (2003). The economics of iron deficiency. Food Policy.

[4] Harrison KA (1988). Severity of Anemia and operative mortality and morbidity. Lancet.

[5] Brabin BJHM, Pelletier D (2001). An analysis of anemia and pregnancy-related maternal mortality. J Nutr.

[6] Kassebaum NJ, Jasrasaria R, Naghavi M, Wulf SK, Johns N, Lozano R (2014). A systematic analysis of global anemia burden from 1990 to 2010. Blood.

[7] World Health Organization. Assessing the iron status of populations: including literature reviews: report of a Joint World Health Organization/Centers for Disease Control and Prevention Technical Consultation on the Assessment of Iron Status at the Population Level, second edition. Geneva: WHO Press; 2004.

[8] Goodenough LT, Nemeth E (2013). Iron deficiency and related disorders. Wintrobe’s clinical hematology 13th Edition.

[9] Allen LHC-SJ, Ramarkrishnan U (2001). Prevalence and causes of nutritional anemias. Nutritional Anemias Boca Raton.

[10] LM MJ, Dalmiya N, Sethuraman K, Gillenwater K (2001). Progress in controlling micronutrient deficiencies.

[11] World Health Organization. Worldwide prevalence of anaemia (1993). WHO global database on anaemia.

[12] Dreyfuss MLSR, Shrestha JB (2000). Hookworms, malaria and vitamin a deficiency contribute to anemia and iron deficiency among pregnant women in the plains of Nepal. J Nutr.

[13] World Health Organization. Global Health Observatory data repository: Prevalence of anaemia in women of reproductive age Estimates by country. 2016. https://apps.who.int/gho/data/node.main.ANEMIA3?lang=en. Accessed 4 Nov 2019.

[14] Sanchaisuriya K, Fucharoen S, Ratanasiri T, Sanchaisuriya P, Fucharoen G, Dietz E (2006). Thalassemia and hemoglobinopathies rather than iron deficiency are major causes of pregnancy-related anemia in Northeast Thailand. Blood Cells Mol Dis.

[15] Karakochuk CD, Whitfield KC, Barr SI, Lamers Y, Devlin AM, Vercauteren SM (2015). Genetic hemoglobin disorders rather than iron deficiency are a major predictor of hemoglobin concentration in women of reproductive age in rural prey Veng. Cambodia J Nutr.

[16] Pansuwan A, Fucharoen G, Fucharoen S, Himakhun B, Dangwiboon S (2011). Anemia, iron deficiency and thalassemia among adolescents in Northeast Thailand: results from two independent surveys. Acta Haematol.

[17] Weatherall DJ, Clegg JB. The biology of the thalassaemias. The Thalassaemia Syndromes, 4th Edition. Wiley-Blackwell; 2008. p. 133-236.

[18] Zimmermann MB, Fucharoen S, Winichagoon P, Sirankapracha P, Zeder C, Gowachirapant S (2008). Iron metabolism in heterozygotes for hemoglobin E (HbE), alpha-thalassemia 1, or beta-thalassemia and in compound heterozygotes for HbE/beta- thalassemia. Am J Clin Nutr.

[19] MacKenzie EL, Iwasaki K, Tsuji Y (2008). Intracellular iron transport and storage: from molecular mechanisms to health implications. Antioxid Redox Signal.

[20] Tangsuwansopin P, Sanchaisuriya P, Sanchaisuriya K, Chaitripop C, Yamsri S, Fucharoen S, Fucharoen G (2014). Anemia, iron deficiency and iron deficiency anemia among female university students in Khon Kaen Province: relationship to menstruation. J Med Tech Phys Ther.

[21] Thompson D, Milford-Ward A, Whicher J (1992). The value of acute phase protein measurement in clinical practice. Ann Clin Biochem.

[22] Northrop-Clewes CA (2008). Interpreting indicators of iron status during an acute phase response – lessons from malaria and human immunodeficiency virus. Ann Clin Biochem.

[23] Yamsri S, Sanchaisuriya K, Fucharoen G, Sae-Ung N, Fucharoen S (2011). Genotype and phenotype characterizations in a large cohort of beta-thalassemia heterozygote with different forms of alpha-thalassemia in Northeast Thailand. Blood Cells Mol Dis.

[24] Sae-ung N, Fucharoen G, Sanchaisuriya K, Fucharoen S (2007). Alpha(0)-thalassemia and related disorders in Northeast Thailand: a molecular and hematological characterization. Acta Haematol.

[25] Sanchaisuriya K, Fucharoen G, Fucharoen S (2002). Hb Pakse (alpha2) codon 142 (TAA-->TAT or term-->Tyr)J in Thai patients with EABart’s disease and Hb H disease. Hemoglobin.

[26] Boonsa S, Sanchaisuriya K, Fucharoen G, Wiangnon S, Jetsrisuparb A, Fucharoen S (2004). The diverse molecular basis and hematological features of Hb H and AEBart's diseases in Northeast Thailand. Acta Haematol.

[27] World Health Organization (2001). Iron deficiency anaemia: Assessment, prevention, and control. A guide for programme managers.

[28] Brimson S, Suwanwong Y, Brimson JM. Nutritional anemia predominant form of anemia in educated young Thai women. Ethn Health. 2017:1–10. 10.1080/13557858.2017.1346188 28669237

[29] Tanphaichitr VS, Mahasandana C, Suvatte V, Yodthong S, Pung-amritt P, Seeloem J (1995). Prevalence of hemoglobin E, alpha-thalassemia and glucose-6-phosphate dehydrogenase deficiency in 1,000 cord bloods studied in Bangkok. Southeast Asian J Trop Med Public Health.

[30] Kadegasem P, Songdej D, Lertthammakiat S, Chuansumrit A, Paisooksantivatana K, Mahaklan L (2019). Reticulocyte hemoglobin equivalent in a thalassemia-prevalent area. Pediatr Int.

[31] Wongprachum K, Sanchaisuriya K, Sanchaisuriya P, Siridamrongvattana S, Manpeun S, Schlep FP (2012). Proxy indicators for identifying iron deficiency among anemic vegetarians in an area prevalent for thalassemia and hemoglobinopathies. Acta Haematol.

[32] Knowles J, Thurnham DI, Phengdy B, Houamboun K, Philavong K, Keomoungkhone I (2013). Impact of inflammation on the biomarkers of iron status in a cross-sectional survey of Lao women and children. Br J Nutr.

[33] Borgna-Pignatti C, Galanello R (2013). Thalassemias and related disorders: Quantitative disorders of hemoglobin synthesis. Wintrobe’s clinical hematology 13th Edition.

[34] Fucharoen S, Winichagoon P (1992). Thalassemia in Southeast Asia: problems and strategy for prevention and control. Southeast Asian J Trop Med Public Health..

[35] Origa R, Galanello R, Ganz T, Giagu N, Maccioni L, Faa G (2007). Liver iron concentrations and urinary hepcidin in beta-thalassemia. Haematol.

[36] Nemeth E (2008). Iron regulation and erythropoiesis. Curr Opin Hematol.

[37] Gardenghi S, Marongiu MF, Ramos P (2007). Ineffective erythropoiesis in beta-thalassemia is characterized by increased iron absorption mediated by down-regulation of hepcidin and up-regulation of ferroportin. Blood.

[38] George J, Yiannakis M, Main B, Devenish R, Anderson C, An US (2012). Genetic hemoglobin disorders, infection, and deficiencies of iron and vitamin a determine anemia in young Cambodian children. J Nutr.

[39] Patel EU, White JL, Bloch EM, Grabowski MK, Gehrie EA, Lokhandwala PM (2019). Association of blood donation with iron deficiency among adolescent and adult females in the United States: a nationally representative study. Transfus.

[40] Harvey LJ, Armah CN, Dainty JR, Foxall RJ, Lewis DJ, Langford NJ, Fairweather Tait SJ (2005). Impact of menstrual blood loss and diet on iron deficiency among women in the UK. Br J Nutr.

[41] Nelson M, Poulter J (2004). Impact of tea drinking on iron status in the UK: a review. J Hum Nutr Diet.

[42] Ahmad Fuzi SF, Koller D, Bruggraber S, Pereira DI, Dainty JR, Mushtaq S (2017). A 1-h time interval between a meal containing iron and consumption of tea attenuates the inhibitory effects on iron absorption: a controlled trial in a cohort of healthy UK women using a stable iron isotope. Am J Clin Nutr.

[43] Cowin I, Emond A, Emmett P, ALSPAC Study Group (2001). Association between composition of the diet and haemoglobin and ferritin levels in 18-month-old children. Eur J Clin Nutr.

[44] Mennen L, Hirvonen T, Arnault N, Bertrais S, Galan P, Hercberg S (2007). Consumption of black, green and herbal tea and iron status in French adults. Eur J Clin Nutr.

[45] Hogenkamp PS, Jerling JC, Hoekstra T, Melse-Boonstra A, MacIntyre UE (2008). Association between consumption of black tea and iron status in adult Africans in the north West Province: the THUSA study. Br J Nutr.

[46] Al-Alimi AA, Bashanfer S, Morish MA (2018). Prevalence of Iron deficiency Anemia among university students in Hodeida Province. Yemen Anemia.

[47] Fan FS (2016). Iron deficiency anemia due to excessive green tea drinking. Clin Case Rep.

[48] Fishman SM, Christian P, West KP (2000). The role of vitamins in the prevention and control of anaemia. Public Health Nutr.

